# The Influence of Diisocyanate Structure on Thermal Stability of Thermoplastic Polyurethane Elastomers Based on Diphenylmethane-Derivative Chain Extender with Sulfur Atoms

**DOI:** 10.3390/ma16072618

**Published:** 2023-03-25

**Authors:** Magdalena Rogulska

**Affiliations:** Department of Polymer Chemistry, Institute of Chemical Sciences, Faculty of Chemistry, Maria Curie-Skłodowska University in Lublin, Gliniana 33, 20-614 Lublin, Poland; mrogulska@umcs.lublin.pl

**Keywords:** thermoplastic elastomer, sulfur-containing chain extender, polyether soft segment, aliphatic diisocyanate, aromatic diisocyanate, thermogravimetry, TG-FTIR

## Abstract

The work is a continuation of the research on thermoplastic polyurethane (TPU) elastomers containing sulfur atoms which are incorporated into the polyurethane chain using aliphatic-aromatic chain extenders. These materials show some improved properties in relation to conventional ones, e.g., adhesion to metals, bacterial resistance and refractive index. The present study deals with the detailed characteristics of the process of thermal decomposition of TPU elastomers obtained from 2,2′-[methylenebis(1,4-phenylenemethylenethio)]diethanol, 1,1′-methanediylbis(4-isocyanatobenzene) (MDI) or 1,6-diisocyanatohexane (HDI) and poly(oxytetramethylene) diol of *M*_n_ = 2000 g/mol by thermogravimetric analysis coupled on-line with Fourier transform infrared spectroscopy. The analysis was performed under inert and oxidative conditions. All TPU elastomers were found to have a relatively good thermal stability, with those based on aromatic diisocyanate being at an advantage. In helium, they are stable up to 280–282 °C (from HDI) and 299–301 °C (from MDI), whereas in synthetic air up to 252–265 °C (from HDI) and 261–272 °C (from MDI), as measured by the temperature of 1% mass loss. Depending on the content of the hard segments and the tested atmosphere, the TPU elastomers decompose from one to four stages. From the analysis of the volatile decomposition products, it follows that the decomposition of both types of hard segments was accompanied by the evolution of carbonyl sulfide, carbon dioxide, water, sulfide dioxide, alcohols and aromatic compounds. For the hard segment derived from HDI, isocyanates, amines, and unsaturated compounds were also identified, while for the MDI-derived one, aldehydes were discovered. In turn, the polyether soft segment decomposed mainly into aliphatic ethers, aldehydes, and carbon monoxide.

## 1. Introduction

The first thermoplastic polyurethanes (TPUs) introduced into the market in the 1940s were linear polymers obtained by the polyaddition reactions of equimolar amounts of diisocyanates and aliphatic diols. These types of polyurethanes, with properties similar to mass-produced polyamides, have a limited application due to higher production costs [1]. Of much more industrial importance were TPU elastomers, which first appeared in commerce twenty years later [1,2]. Currently, they are used, among others, in medicine (prostheses of blood vessels and joints, elements of artificial heart, diaphragms for dialysis, infusion pumps), automotive (airbags, ABS brake systems), machine (sleeves, couplings), electrotechnical (tires of flexible cables), textile and footwear (soles of shoes, foils to cover knitted fabrics) industries [2,3,4,5,6,7,8,9]. In the solid state, they exhibit properties typical of elastomers, while at elevated temperature they plasticize and can be processed using the methods utilized for thermoplastics [2,3,10,11]. The possibility of re-processing has become one of the reasons for the universality of their application. Other reasons include their unique properties combining high tensile strength with high resistance to dynamic loads and a large modulus of elasticity with high elongation at break as well as high resistance to fatigue, low abrasion, very good resistance to weather conditions, oils, mineral greases and fats [10,12,13,14]. The useful characteristics of TPU elastomers are due in part to their segmented structure. They are multiblock copolymers, whose macromolecules comprise alternating hard segments, built from diisocyanate and short chain extender (usually a diol) as well as soft segments formed by an oligomer diol. Selecting the appropriate reactant components is key to achieving TPU elastomers with the desired properties to meet customer requirements. The oligoester diols generally give materials of a higher tensile strength, harder and less stretchable than oligoether diols which are moreover more resistant to ultraviolet rays and oxidation. However, from the oligoether diols polymers exhibiting improved low-temperature properties and better resistance to high humidity conditions as well as attack by fungi and bacteria are synthesized [2,15,16]. As for the isocyanate component, it is well known that aromatic diisocyanates are used to obtain polymers of high tensile strength and modulus of elasticity, as well as thermal resistance [17,18,19]. Unluckily, these polymers tend to yellow when exposed to heat or light because of the formation of quinone-imide structures, limiting their applications for external use. In turn, aliphatic and cycloaliphatic diisocyanates allow to obtain materials resistant to both light and heat, characterized also by a greater degree of microphase separation and improved resistance to hydrolytic degradation, often at the expense of deterioration of mechanical properties [17,19,20,21,22]. Similar to diisocyanates, chain extenders can contain aliphatic or aromatic units. Using aliphatic-aromatic and aromatic diols mostly yields harder polyurethanes of a higher modulus of elasticity than from aliphatic diols. On the other hand, from among these “bulky” diols, aliphatic-aromatic ones produce polyurethanes with higher elongation at break and slightly lower hardness [2,10,23]. Both aromatic and aliphatic-aromatic diols are employed to synthesize polyurethanes characterized by improved thermal stability [24,25,26] or liquid-crystalline properties [27] as well. The introduction of bromine atoms into the structure of these diols makes it possible to additionally improve the flame retardancy of the materials obtained [28].

Some interesting properties of TPU elastomers can also be enhanced by introducing sulfur atoms into the structure of “bulky” diols [29,30,31,32,33,34,35] or alternatively by using “bulky” dithiols [36,37,38,39]. From the literature dealing with sulfur-containing polymers it follows that the presence of sulfur atoms can enhance adhesion to metals, chemical and microbiological resistance, refractive index, self-healing properties, flexibility as well as microphase separation and lower-temperature properties of such materials compared to conventional ones [40,41,42,43,44,45,46,47,48,49]. Research conducted for TPU elastomers based on aliphatic-aromatic diols with sulfide linkages or dithiols showed that it is possible to obtain materials combining good thermal stability and mechanical properties with greater adhesion strength and/or refractive index [29,30,31,32,36,37]. Moreover, the applied manner of modification of TPU elastomers by means of such chain extenders simplifies the technological process and allows to obtain products with desirable properties already at the polymer formation stage without adding other substances. Recently [50], a preliminary study on the effect of using a nonconventional sulfur-containing diol on the antimicrobial activity of the TPU elastomers based on aliphatic or aromatic diisocyanate were also conducted. For the study, new polymers were selected which were synthesized from diphenylmethane-derivative diol, i.e., 2,2′-[methanediylbis(benzene-1,4-diylmethanediylsulfanediyl)]diethanol, designated as DPHM-E diol, 1,1′-methylenebis(4-isocyanatobenzene) (MDI) or 1,6-diisocyanatohexane (HDI) and poly(oxytetramethylene) diol (PTMO) with the molar mass of 2000 g/mol. Because of difficult access to the results obtained, a brief summary is given below. Screening tests of antimicrobial activity performed for TPU elastomers with the 40 mas% hard-segment content showed that the presence of these polymers inhibited the proliferation of Gram-positive bacteria (*Staphylococcus epidermidis*, ATCC 12228), with much better effects observed for the HDI-based one. This polymer also reduced the number of Gram-negative colonies (*Escherichia coli*, ATCC 25922). In the case of fungi (*Candida albicans*, ATCC 10231), the introduction of TPU elastomers into the culture medium did not significantly affect their growth. The density of cultures in the presence of and without the addition of the polymer (strain proliferation control) was measured at 48 and 72 h and after seven days of incubation. Moreover, polymers of both types were found to exhibit about twice the adhesion strength to copper in comparison with their counterparts synthesized from butane-1,4-diol. Nevertheless, better adhesive properties were shown by the MDI derivative. For all obtained materials, i.e., those containing 30–60 mass% of hard segments, the structure and some basic properties were determined using atomic force microscopy, differential scanning calorimetry, tensile tests, and Shore hardness. Both the HDI and MDI polymers were colorless materials with high molar masses. They had partially ordered structures, within both the hard-segment and soft-segment domains. However, much better ordered hard segments were revealed by the MDI polymers. In turn, the TPU elastomers of the HDI series exhibited lower glass-transition temperatures (up to −73 °C vs. −64 °C) and a higher degree of microphase separation compared to those of the MDI series. The analysis of mechanical properties showed that polymers with lower Shore hardness and elastic modulus but higher tensile strength (up to 26.0 MPa vs. 21.3 MPa) and higher elongation at break (up to 750% vs. 640%) were synthesized from MDI than from HDI. Based on the results, it can be concluded that the use of a polyether soft segment with a higher molar mass positively influenced the degree of microphase separation of TPU elastomers and in most cases also their mechanical strength [51,52]. The results provided encouragement to continue research on these polymers.

The objective of the present paper was to determine the thermal stability of the TPU elastomers described above using a dynamic method based on thermogravimetry (TG) coupled on-line with Fourier transform infrared (FTIR) spectroscopy. This method is a useful tool as it monitors continuously both the temperature dependent evolution of the volatiles and the mass of the residual substances.

The analysis of the thermal decomposition of TPU elastomers is important not only from the fundamental point of view, but also from the technological one. This is because it allows to obtain information on the optimal conditions for designing and processing these thermoplastic materials. In addition, thermal decomposition research provides information used in the safe disposal or recycling of polymer wastes. It should be stressed that at elevated temperatures, the thermal stability of such complex systems as polyurethanes need not be affected by the weakest link in the chain, but is often influenced by the environment [53,54]. So, the experiments were carried out in inert (helium) and oxidative (synthetic air) atmospheres.

## 2. Materials and Methods

### 2.1. Materials

The DPHM-E diol (m.p. = 77–78 °C) was synthesized by the reaction of (methanediyldibenzene-1,4-diyl)dimethanethiol with 2-chloroethanol in a water-ethanolic sodium hydroxide solution and crystallized from benzene [52]. Sodium hydroxide, ethanol and benzene (POCh, Gliwice, Poland) and 2-chloroethanol (Fluka, Buchs, Switzerland) were analytical reagent grade and used as received. Moreover, the reagents, such as MDI (98%) from Sigma-Aldrich (Steinheim, Germany), HDI (99%) and dibutyltin dilaurate (DBTDL) from Merck Schuchardt (Hohenbrunn, Germany), were used without further purification. In turn, prior to use, PTMO with the molar mass of 2000 g/mol from Sigma-Aldrich (Germany) was heated at 90 °C in vacuo for 10 h.

### 2.2. Synthesis of TPUs

**Figure 1:**
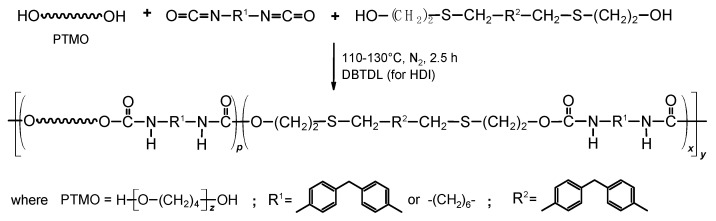
Schematic representation of the synthesis route of TPU elastomers.

In accordance with the scheme in Figure 1 two series of TPU elastomers with the hard-segment content of 30, 40, 50 and 60 mas% were obtained by using the diphenylmethane-derivative diol (DPHM-E diol) and MDI or HDI as components of the hard segments and PTMO as a soft segment. In case less reactive aliphatic diisocyanate was used, syntheses were carried out in the presence of DBTDL as a catalyst. 

The syntheses were carried out using a solvent-free one-step polyaddition procedure under dry nitrogen as previously reported [50]. To synthesize the HDI-based TPU elastomers, the DPHM-E diol and PTMO (0.01 mol together) and HDI (0.0105 mol) were weighed out into a three-necked round-bottom flask fitted with a mechanical stirrer, a gas inlet tube and a calcium chloride drying tube, and placed in an oil bath of a temperature 110 °C. Next to the clear and mixed melt DBTDL (0.03 g) was added and polymerization rapidly began at vigorous stirring. In the case of the MDI-based TPU elastomers, diisocyanate (0.01 mol) was put into a flask only after the melting and mixing of dihydroxy compounds at 110 °C. The reactions were continued until the viscosity increase made stirring impossible. Then the reaction temperature was gradually raised to 130 °C and the formed colorless rubber-like products were conditioned at this temperature for 2 h.

The syntheses of hard-segment-type TPUs were performed in the same way as the TPU elastomers.

Designations of the TPUs are given in Table 1. As can be seen the TPUs were designated as X-Y, where X is the abbreviation of the diisocyanate and Y represents the content of hard segments. The hard-segment content was calculated as the ratio of the mass of a diisocyanate and the DPHM-E diol to the total mass of the polymer.

### 2.3. Measurement Methods

A Bruker Tensor 27 FTIR spectrometer (Hanau, Germany) with attenuated total reflectance (ATR) module was used to acquire all the FTIR spectra. The instrument acquisition parameters were set to absorbance mode with 32 scans per spectrum, 4 cm^−1^ resolution and the spectral range of 600 and 4000 cm^−1^. 

TG measurements were done on a Netzsch STA 449 F1 Jupiter thermal analyzer (Selb, Germany) in flowing at 20 cm^3^/min helium or synthetic air (80% nitrogen, 20% oxygen). The samples were heated from 30 °C to 600 °C (in helium atmosphere) or 700 °C (in synthetic air atmosphere) using the heating rate of 10 °C/min. The samples of 10 ± 0.2 mg were placed in the open Al_2_O_3_ crucibles (mass of about 140 mg). As a reference, an empty Al_2_O_3_ crucible was applied. From the registered TG curves the temperatures of 1% (*T*_1%_), 5% (*T*_5%_), 10% (*T*_10%_) and 50% (*T*_50%_) mass losses were determined. In turn, the temperature of the maximum rate of mass loss (*T*_max_) for particular decomposition stages was designed on the basis of differential TG (DTG) curves. Mass loss responding to a given stage of decomposition was also read. These parameters were collected in Table 1 and Table 2. In the case of one-stage decomposition, one *T*_max_ and one mass loss were reported, while in the case where polyurethane decomposition occurred in two or three stages, two or three *T*_max_s were reported, along with the corresponding (two or three) mass losses.

The composition of gases evolved during the decomposition process was detected and analyzed by a Bruker Tensor 27 FTIR spectrometer (Germany) coupled on-line to a Netzsch STA instrument. The transfer line to the FTIR spectrometer was made from teflon, had an interior diameter of 2 mm and was heated to 200 °C. The FTIR spectra acquired with a spectral resolution of 4 cm^−1^ on 600–4000 cm^−1^ range. Sixteen spectral scans were conducted on each sample.

## 3. Results and Discussion

**Figure 2:**
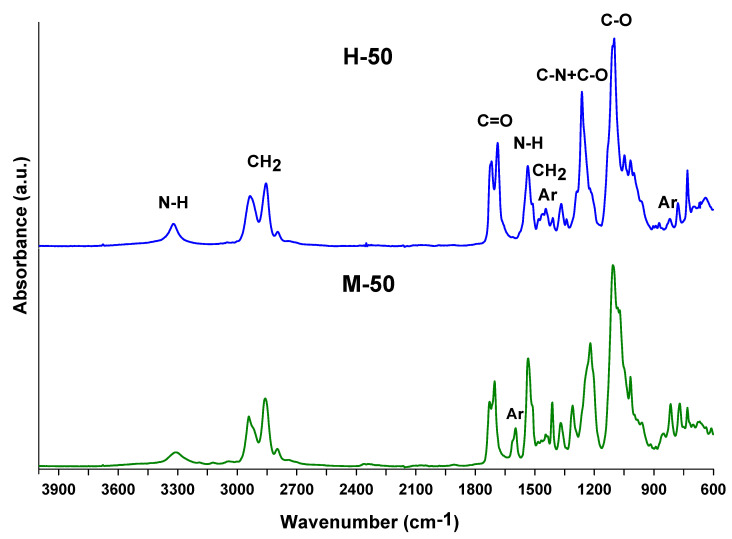
ATR-FTIR spectra of the H-50 and M-50 TPU elastomers.

The chemical structures of the TPUs were verified by ATR FTIR spectroscopy. All the spectra reveal the absorption bands of the urethane and methylene groups as well as the benzene ring. Absorption bands confirming the presence of the ether group are also visible for TPU elastomers. In turn, the absence of a band at approximately 2270–2260 cm^−1^ indicates that all isocyanate groups were converted to urethane ones. The major absorption bands are given below, whereas representative spectra of TPU elastomers (M-50 and H-50) are shown in Figure 2. 

ATR-FTIR (cm^−1^): 3321–3305 (N-H stretching), 1731–1683 (C=O stretching), 1536–1535 (N-H bending) and 1247-1221 (coupled C-N and C-O stretching) of the urethane group; 1107–1100 (C-O stretching of the ether group); 2941–2935 and 2857–2854 (asymmetric and symmetric C-H stretching of the methylene group); 1481–1446 (C-H bending of methylene group and C-C stretching of the benzene ring); 1598–1597 (C-C stretching of the benzene ring for TPUs based on MDI); 817–816 (C-H bending of *p*-disubstituted benzene ring). 

**Figure 3:**
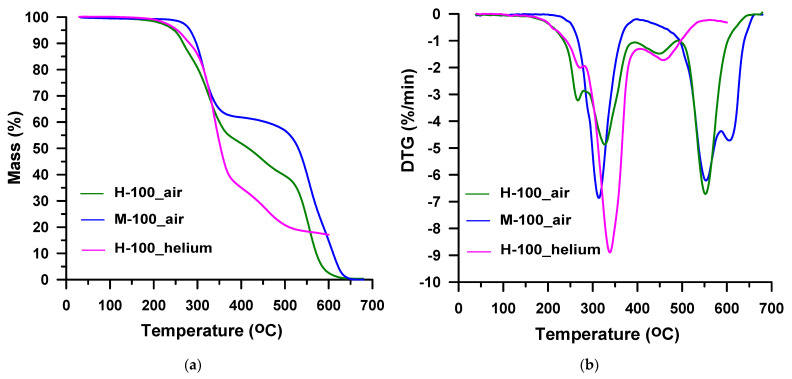
TG (**a**) and DTG (**b**) curves of the hard-segment-type TPUs obtained in helium and air.

Thermal decomposition of polyurethanes generally is a multi-stage process, both in inert and oxidative conditions. As a result, various gaseous products are created, the analysis of which is possible using the FTIR spectrometer. Firstly, the TG-FTIR analysis was made for TPUs H-100 and M-100, which constitute the patterns of hard segments in these elastomers. In the case of the H-100 sample, the tests were carried out both in inert and oxidative atmospheres, while for the M-100 sample only in an oxidative one. The numerical data obtained are summarized in Table 1 and Table 2, while the TG-DTG curves are presented in Figure 3. Tests in an inert atmosphere (argon) for the H-100 sample were performed earlier and presented in a previous paper [52]. The received numerical data, however, was posted in Table 1 to facilitate the interpretation of the results obtained for the TPU elastomers.

**Table 1:** Designations and TG-DTG data obtained for TPUs in the helium atmosphere.

TPU	Hard-Segment Content (Mas%)	Diisocyanate	*T*_1%_ (°C)	*T*_5%_ (°C)	*T*_10%_ (°C)	*T*_50%_ (°C)	*T*_max_ (°C)	Mass Losses (%)
H-30	30	HDI	281	322	341	388	398	99
H-40	40		280	316	335	383	370, 398	38, 61
H-50	50		282	315	334	380	362, 396	41, 58
H-60	60		282	313	328	370	357, 393	60, 39
H-100	100		203	254	282	353	272, 338, 458	10, 56, 17
M-30	30	MDI	301	333	346	383	370, 391	36, 62
M-40	40		301	331	344	382	364, 391	44, 53
M-50	50		300	328	340	378	361, 388	47, 48
M-60	60		299	328	339	377	359, 385	53, 41
M-100 *	100		259	315	344	380	373, 521	-

* Data obtained in the argon atmosphere [52].

**Table 2:** TG-DTG data obtained for TPUs in the synthetic air atmosphere.

TPU	*T*_1%_ (°C)	*T*_5%_ (°C)	*T*_10%_ (°C)	*T*_50%_ (°C)	*T*_max_ (°C)	Mass Losses (%)
H-30	265	306	324	382	346, 382, 530	27, 57, 15
H-40	264	306	322	387	336, 393, 537	28, 54, 17
H-50	262	307	321	392	332, 397, 543	30, 52, 17
H-60	252	302	315	386	330, 389, 541	42, 32, 25
H-100	180	246	267	413	267, 327, 450, 552	14, 33, 12, 41
M-30	272	320	333	352	340, 392, 540	23, 51, 20
M-40	270	310	325	403	335, 399, 549	25, 49, 26
M-50	268	311	331	395	336, 355, 393, 529	13, 25, 31, 31
M-60	261	304	320	401	326, 359, 392, 545	19, 19, 24, 38
M-100	237	283	297	529	314, 553, 605	38, 42, 20

Taking into consideration the value of *T*_1%_, it should be said that the hard-segment-type TPU derived from an aliphatic diisocyanate is stable up to 203 °C in the helium atmosphere. In the air atmosphere, the value of this indicator is lower by 23 °C. In the case of the remaining parameters determining the initial stage of decomposition, the differences between the inert and oxidative atmospheres are smaller. Thus, *T*_5%_ is 254 °C vs. 246 °C and *T*_10%_ is 282 °C vs. 267 °C. It proves the unfavorable effect of oxidation processes on its thermal stability. The deterioration of stability in the air is also observed for the M-100 sample. Nevertheless, all the mentioned indicators are higher for the M-100 sample in comparison with the H-100 one, both in the inert and oxidative conditions. The differences in the *T*_1%_, *T*_5%_ and *T*_10%_ values are ~55–60 °C in helium and ~30–55 °C in synthetic air. Also, the values of *T*_50%_ are higher for this polymer. This points to a better thermal stability of TPU derived from an aromatic diisocyanate in relation to its analog obtained from an aliphatic one. It is a well-known fact that the resistance to the thermally activated oxidation of polyurethanes from aromatic diisocyanates is higher than that from aliphatic ones, regardless of the darkening of the former. The oxidation process is strongly accelerated by organometallic tin compounds, such as DBTDL used in the synthesis of the H-100 sample as a catalyst [10,54]. When it comes to thermal stability in an inert atmosphere, polyurethanes based on aliphatic diisocyanates are generally more resistant. The uncommon behavior of the studied polymers may result from better ordering [10,54] of the M-100 sample compared to the H-100 one. Such a relationship was previously found for similar hard-segment-type TPUs based on diphenylethane-derivative diol [55].

The DTG curve obtained for the H-100 sample in the atmosphere of helium (Figure 3b) reveals three, partly overlapping peaks with maxima at 272, 338 and 458 °C. Although these peaks are not well-separated, it is possible to approximate the mass losses corresponding to the particular decomposition steps. Thus, the peak with the highest intensity (*T*_max_ = 338 °C) relates to 56% mass loss. For the remaining two peaks the mass losses are 10 and 17%, respectively. In the case of the DTG curve obtained for the H-100 sample in the air atmosphere (Figure 3b), four peaks are observed, which also partially overlap each other. This four-stage decomposition has *T*_max_s of 267, 327, 450 and 552 °C. The largest mass loss of 41% occurs in the fourth stage. The course of decomposition of TPU M-100 in the air atmosphere (Figure 3b) differs from the course of decomposition of its analog derived from HDI. It does not take place in four stages, but only in three. The first stage is observed in the temperature range of 240–395 °C (with a maximum at 314 °C) with 38% mass loss. It is well separated from the other two stages which overlap. The second and third ones spread from 395 to 670 °C (with maxima of 553 and 605 °C) and have a total mass loss of 62%.

**Figure 4:**
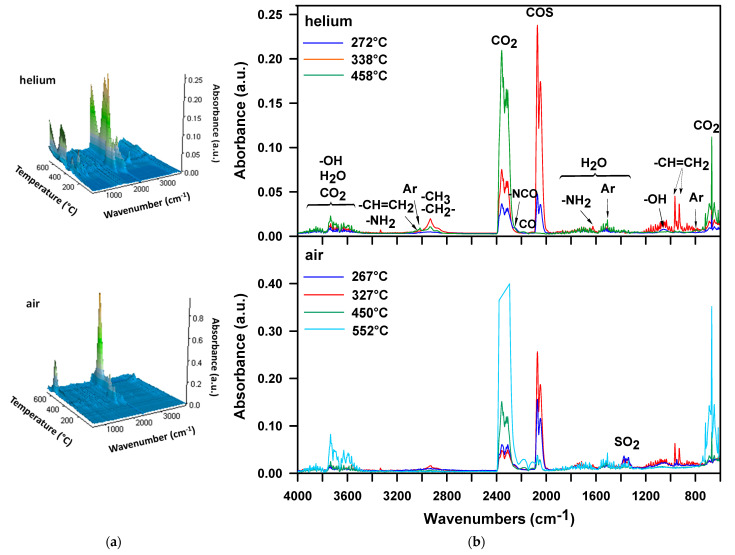
FTIR spectra of volatile products evolved during thermal decomposition of H-100: (**a**) 3D and (**b**) extracted at the maxima of decomposition.

The FTIR spectrum recorded during the first decomposition step of the H-100 sample in helium (Figure 4b, *T*_max_ = 272 °C) shows bands typical of carbonyl sulfide (at 2072 and 2047 cm^−1^, characteristic of the asymmetric and symmetric C=O stretching vibrations, respectively), carbon dioxide (at 2359–2310 cm^−1^, connected with asymmetric stretching vibrations and at 669 cm^−1^—with the degenerate bending vibrations), primary alcohols (at 1055 cm^−1^, related to the C-OH stretching vibrations) and water (at ~4000–3500 and ~1800–1300 cm^−1^, associated with stretching and bending vibrations, correspondingly). The bands in the ~4000–3500 range also come from carbon dioxide (combination bands) and alcohols (the O-H stretching vibrations). Moreover, a band pointing to the presence of an isocyanate (at 2260 cm^−1^, characteristic of the asymmetric N=C=O stretching vibrations) is visible. The formation of alcohols found in the decomposition products of the DPHM-E diol [52] and isocyanate proves that the decomposition of the urethane bonds is connected with their dissociation. Nevertheless, the low intensity of the band at 2260 cm^−1^ is probably due to partial carbodiimidization of an isocyanate [54,56]:

R^1^NHCOOR^2^ → R^1^NCO + R^2^OH

2R^1^NCO → R^1^-N=C=N-R^1^ + CO_2_

According to the literature [57], from the temperature of 320 °C, carbodiimides degrade to produce isocyanates. Since isocyanates are not observed among the detected volatile substances above this temperature, one can assume that the isocyanate groups reacted with a water molecule to give a less volatile urea compound.

During the second step (Figure 4b, *T*_max_ = 338 °C), carbonyl sulfide, carbon dioxide and water are still the main decomposition products. There are no bands showing the creation of isocyanate and alcohols. However, in this stage, aliphatic unsaturated hydrocarbons and amines are emitted. This is evidenced by the following bands: at 3025 cm^−1^ as well as at 965 and 931 cm^−1^, attributed to the C-H stretching and out-of-plane deformation vibrations, respectively, of the vinyl group and at 2933 and 2855 cm^−1^, related to the asymmetric and symmetric C-H stretching vibrations, respectively, of the methylene and methyl groups and at 3334 and 1625 cm^−1^, associated with N-H stretching and bending vibrations, respectively, of the amine group. Thus, it can be assumed that the decomposition of the urethane bonds also occurs with the elimination of carbon dioxide, according to the following reaction [54,56,58]:

R^1^NHCOOCH_2_CH_2_R^2^ → R^1^NH_2_ + CO_2_ + CH_2_=CHR^2^

**Figure 5:**
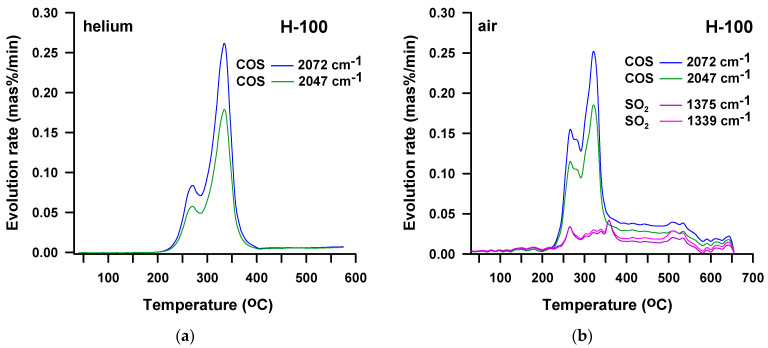
(**a**) Carbonyl sulfide release for H-100 sample during TG-FTIR analysis in helium and (**b**) carbonyl sulfide and sulfur dioxide release for H-100 sample during TG-FTIR analysis in air.

In turn, the FTIR spectrum from the third decomposition step (Figure 4b, *T*_max_ = 458 °C) displays mainly bands pointing to the presence of carbon dioxide, carbon monoxide (at 2175 cm^−1^, attributed to the stretching vibrations) and aromatic compounds (at 3027 and 1508 cm^−1^, associated with the C-H and C-C stretching vibrations, correspondingly). It should be emphasized that in the second stage the most carbonyl sulfide is created, which indicates that the main decomposition of the sulfide bonds occurs in this stage. This also results from the 3D spectrum recorded over the entire measurement temperature range (Figure 4a). The carbonyl sulfide traces (2072 and 2047 cm^−1^) singled out from this spectrum (Figure 5a) show two peaks and the considerably higher is that observed exactly at 338 °C, i.e., at the *T*_max_ of second decomposition step. Considering the 3D spectrum, it is also clear that the decomposition of the urethane bonds starts at a lower temperature than the decomposition of the sulfide ones. This is because the emission of carbonyl sulfide begins at a higher temperature (~205 °C) than carbon dioxide (~180 °C). 

The FTIR spectra obtained from the analysis of the H-100 sample in the air atmosphere (Figure 4) indicate the formation of the products observed in an inert one, i.e., carbonyl sulfide, carbon dioxide, carbon monoxide, water, alcohols, isocyanate, amine as well as aliphatic unsaturated and aromatic compounds. Similar to the decomposition in helium, alcohols and isocyanate are released during the first stage, whereas amines and unsaturated compounds are formed during the second one. Therefore, it should be assumed that the decomposition of the urethane bonds proceeds according to the same mechanisms. The new product detected is sulfur dioxide. Its creation is manifested by the bands at 1375–1339 cm^−1^, associated with the asymmetric S=O stretching vibrations. As can be seen in Figure 5b, the range of carbonyl sulfide release is mostly the same as that of sulfur dioxide. However, the largest amounts of sulfur dioxide are formed at 264 and 362 °C, and carbonyl sulfide at 264 and 323 °C. Thus, it can be said the decomposition of sulfide bonds in an oxidizing atmosphere is accompanied by the simultaneous evolution of two kinds of sulfur-containing oxides. Sulfur dioxide is probably formed by the reaction of carbonyl sulfide with oxygen present in the air [59]. In the third and fourth stages of decomposition, the main products that are evolved are carbon dioxide, carbon monoxide and water vapor. These gaseous products confirm the occurrence of the oxidation reactions of a char residue formed after the first and second decomposition stages. Nevertheless, in the fourth stage, several times more carbon dioxide and carbon monoxide are emitted than in the third one. This means that the main oxidative processes take place above 500 °C.

**Figure 6:**
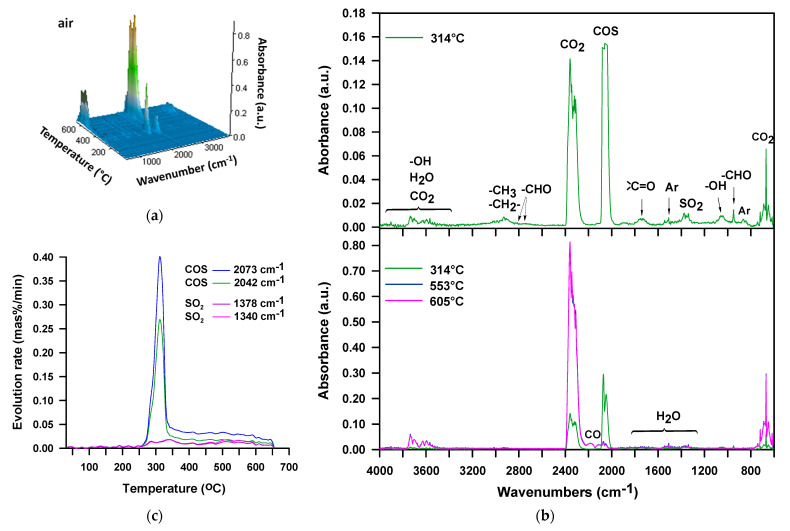
FTIR spectra of volatile products evolved during thermal decomposition of M-100 in air: (**a**) 3D and (**b**) extracted at the maxima of decomposition, (**c**) carbonyl sulfide and sulfur dioxide release for M-100 during TG-FTIR analysis in air.

**Figure 7:**
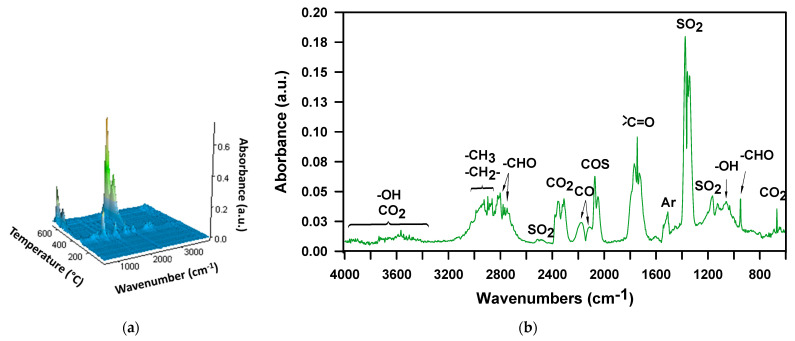
FTIR spectra of volatile products evolved during thermal decomposition of the DPHM-E diol in air: (**a**) 3D and (**b**) extracted at *T*_max_ = 313 °C.

In the case of the M-100 sample decomposition in the air atmosphere, the FTIR spectrum collected at *T*_max_ = 314 °C (Figure 6a,b), just as for the H-100 sample, exhibits bands confirming the existence of carbonyl sulfide, sulfur dioxide, carbon dioxide, water, aliphatic alcohols and aromatic compounds. There are no noticeable bands that signal the formation of isocyanate, amine, and unsaturated compounds. Based on the presence of alcohols, it can be assumed that the urethane bonds are broken down by dissociation here as well. In turn, the absence of bands coming from amine and unsaturated compounds points to the fact that the urethane bonds decomposition does not follow mechanisms that generate primary amines, carbon dioxide and alkenes. There is also no band indicating the presence of ethylene oxide (at 916-912 cm^−1^), detected in the decomposition products of this polymer in an inert atmosphere [52]. Thus, the occurrence of simultaneous decomposition of urethane and sulfide bonds, which takes place with the forming thiourethane intermediaries and followed by the elimination of carbonyl sulfide, cannot be unequivocally confirmed. On the other hand, the spectrum displays the bands characteristic of organic carbonyl compounds, including aldehydes (at ~1730 cm^−1^, the C=O stretching vibrations, at 2814 and 2740 cm^−1^, C-H stretching vibrations and at 950 cm^−1^, C-H bending vibrations). These products are observed among the decomposition volatiles of the DPHM-E diol in the air. Figure 7 illustrates the FTIR spectra of volatile products which evolved during the thermal decomposition of this diol, both in the whole temperature range of the measurement (3D spectrum) and at the temperature of the maximum rate of mass loss for the second (main) stage, i.e., 313 °C. 

Taking into account the course of carbonyl sulfide and sulfide oxide evolution presented in Figure 6c, it can be said that as in the case of the H-100 sample, these volatiles are formed in almost the same temperature range. It should also be noted that the carbonyl sulfide release maximum is consistent with the *T*_max_ of first decomposition step, i.e., 314 °C.

**Figure 8:**
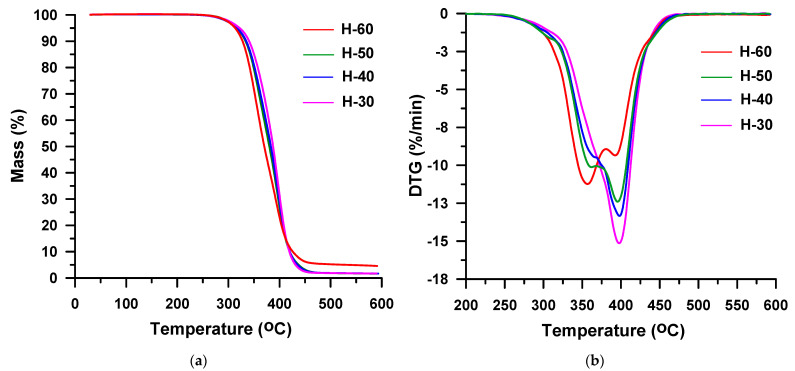
TG (**a**) and DTG (**b**) curves of the HDI-based TPU elastomers obtained in helium.

**Figure 9:**
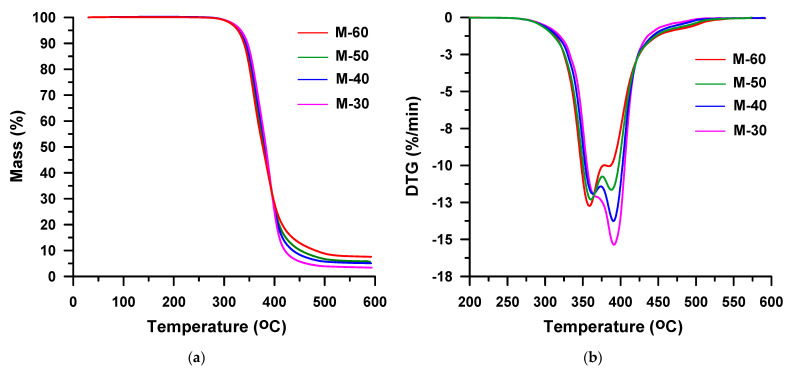
TG (**a**) and DTG (**b**) curves of the MDI-based TPU elastomers obtained in helium.

It is commonly known that in the case of the polyether-based TPUs the urethane group is less resistant to thermal decomposition than the ether group under inert conditions. Thus, the starting temperature of decomposition is determined by the type of isocyanate and alcohol building the urethane group. From the TG data received in helium (Table 1, Figure 8a and Figure 9a), it results that the TPU elastomers synthesized from MDI show higher *T*_1%_, *T*_5%_ and *T*_10%_ than those from HDI, as it was stated for the hard-segment-type TPUs. Moreover, in this case, it is to do with a greater degree of ordering within the hard segments of polymers with MDI as compared to their HDI counterparts. It should be noted that in both series, the content of hard segments has an insignificant influence on the beginning of thermal decomposition. The *T*_1%_s are almost the same and are: 280–282 °C for the HDI series and 299–301 °C for the MDI series. The greater effect of hard-segment contents is visible for the rest of temperature parameters. They increase as the content of hard segments decreases due to the better thermal stability of the polyether soft segment than those of the hard segments. As the HDI-based hard segment is less thermally stable than the MDI-based one, greater differences in *T*_5%_, *T*_10%_ and *T*_50%_ values are seen in the HDI series in relation to the MDI series. Namely, *T*_5%_s are between 313 and 322 °C vs. 328 and 333 °C, *T*_10%_s are between 328 and 341 °C vs. 339 and 346 °C, and *T*_50%_s are between 370 and 388 °C vs. 377 and 383 °C. A drop of stability with increasing content of hard segments was also discovered for TPU elastomers containing a PTMO soft segment that have been derived from MDI and 3,3′-[methylenebis(1,4-phenylenemethylenethio)]dipropan-1-ol [60], 2,2′-[sulfanediylbis(benzene-1,4-diylsulfanediyl)]diethanol [61] or butane-1,4-diol [62]. Taking into account the values of the above-mentioned indicators, it can be said that TPU elastomers under study show relatively good thermal stabilities as for polyurethanes [10,56]. Moreover, they exhibit higher [63,64,65,66] or similar [62,67] values of these parameters than the corresponding poly(ether-urethane)s synthesized from aliphatic or aromatic diisocyanate and aliphatic chain extenders, such as propane-1,3-diol or butane-1,4-diol.

On the DTG curves obtained in the helium atmosphere for the HDI-derived TPU elastomers with high hard-segment contents (H-40, H-50 and H-60) two partly covering peaks are visible (Figure 8b). They show maxima in the range of 357–370 °C and 393–398 °C and indicate a two-stage decomposition of these polymers. The higher content of hard segments in the polymer, the greater the intensity of the lower-temperature peak and the lower the higher-temperature peak intensity. The first stage of decomposition corresponds to mass losses increasing from 38% to 60%, and the second one relates to mass losses decreasing from 61% to 39%. In turn, the DTG curve received for the H-30 sample (Figure 8b) proves its one-stage decomposition. One peak with a *T*_max_ of 398 °C is seen.

The thermal decomposition of all the TPU elastomers based on MDI runs in two overlapped stages, which are seen on DTG curves (Figure 9b). *T*_max_s determined for these polymers are similar to those of the HDI-based derivatives: 359–370 °C and 385–391 °C. Here, too, the increase of the hard-segment-content causes an increase of the peak intensity at lower temperature, and a decrease at higher temperature. Thus, from 36% (for M-30) to 53% (for M-60), mass is lost in the first decomposition stage, whereas in the second decomposition one it is in the range from 62% (for M-30) to 41% (for M-60). Therefore, it can be assumed that in both series the first step is connected with the hard-segment decomposition, while the second one with the soft-segment decomposition. Moreover, it can be said that up to the temperature of 600 °C TPU, elastomers synthesized from MDI decompose to a lesser extent, for which 2–6% residues are found, than those obtained from HDI with 1% residues. This is due to the higher content of benzene rings in the former samples.

**Figure 10:**
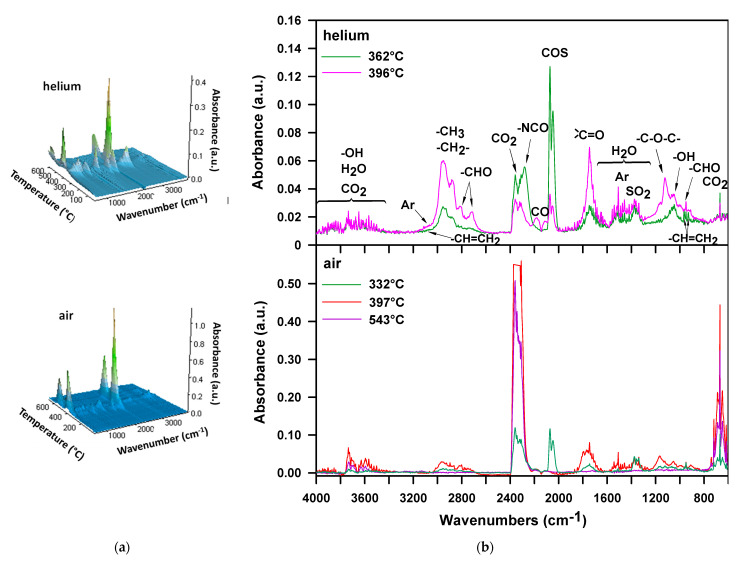
FTIR spectra of volatile products evolved during thermal decomposition of H-50: (**a**) 3D and (**b**) extracted at the maxima of decomposition.

**Figure 11:**
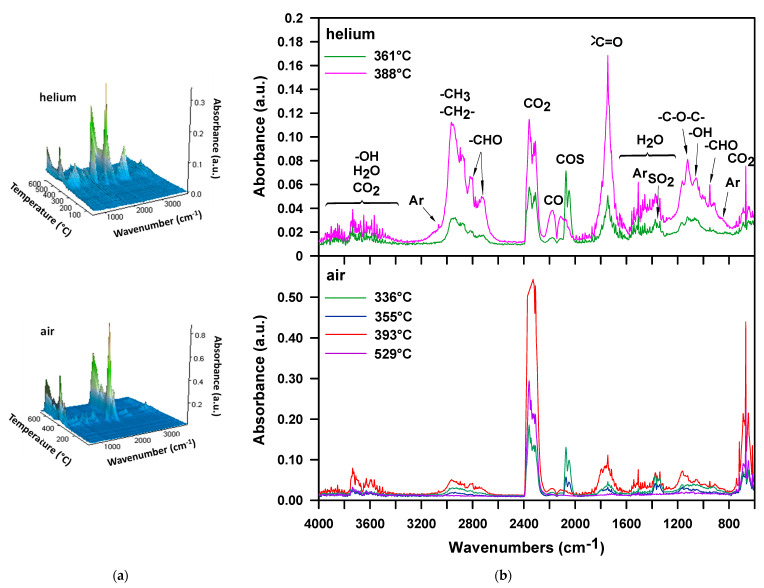
FTIR spectra of volatile products evolved during thermal decomposition of M-50: (**a**) 3D and (**b**) extracted at the maxima of decomposition.

TG-FTIR analysis performed on two selected TPU elastomers (H-50 and M-50) in helium shows that the decomposition of both these polymers is connected with the evolution of almost the same products (see Figure 10 and Figure 11). Namely, the FTIR spectra show the bands confirming the creation of carbonyl sulfide, carbon dioxide, carbon monoxide, water, aliphatic aldehydes, alcohols, ethers and aromatic compounds. In addition, for the H-50 sample, bands from isocyanate and vinyl groups are observed in the first stage of decomposition, which were also discovered for the H-100 sample. So, it can be said that the urethane bonds in this polymer dissociate into alcohol and isocyanate. Due to the lack of distinct bands indicating the formation of amines, it can be assumed that they do not decompose according to the mechanism related to the elimination of carbon dioxide. As for the M-50 sample, one should say that the breakdown of the urethane bonds also occurs only as a result of their dissociation. It should be added that the decomposition of the sulfide bonds in helium is accompanied by the emission of not only carbonyl sulfide but also sulfur dioxide. The formation of sulfur dioxide may be due to oxidative processes caused by oxygen-containing radicals that are produced during the decomposition of PTMO or by certain level of oxygen impurities in the inert purge gas used [68]. Furthermore, a comparison of the FTIR spectra obtained for these TPU elastomers with those of the corresponding hard-segment-type ones shows that the aliphatic ethers and aldehydes originate from the decomposition of PTMO soft segment. As shown in the literature, in inert conditions PTMO can decompose to tetrahydrofuran [54,69], dibutyl ether, butanal and carbon monoxide [70] and many other substances, e.g., butano-1,4-diol, butan-1-ol, 1-propoxybutane and 4-butoxybut-1-ene [62]. From the obtained FTIR spectra is also evident that much more carbonyl sulfide is released in the first decomposition step, which indicates that the hard segments decompose mainly in this step. On the other hand, the decomposition of the soft segment occurs mostly in the second step, as manifested by the greater amount of evolved organic carbonyl compounds, among others, aldehydes, as well as the presence of ethers. This confirms the assumptions made on the basis of the appearance of the TG-DTG curves.

As for thermal stability of the studied TPU elastomers in the air atmosphere, it deteriorates in both series, which is influenced by susceptibility to oxidation. Such observation can also be seen for other TPU elastomers [11,32,67]. From the data contained in Table 1, it follows that all the studied elastomers exhibit lower *T*_1%_, *T*_5%_ and *T*_10%_. The largest differences between the helium and air atmospheres are observed for *T*_1_ and increase with the increase in the content of hard segments. In the HDI series, they are 16–30 °C and in the MDI one 29–38 °C. It can therefore be concluded that the hard segments have a greater impact on the start of thermal decomposition of TPU elastomers than the polyether soft segment which is known not to be oxidative resistant [69]. In the case of *T*_5%_ the differences are 8–24 °C and for *T*_10%_ they are 9–19 °C. However, the tested TPU elastomers derived from MDI are more thermooxidatively stable as compared to polyurethane elastomers obtained from PTMO with the molar mass of 2000 g/mol, MDI or 1,4-diisocyanatobenzene and aliphatic (butane-1,4-diol or 2,2-dimethylpropane-1,3-diol) or aromatic (4,4′-(propane-2,2-diyl)diphenol) chain extender [71]. Moreover, in the case of the HDI series TPU elastomers is can be said that they are more thermooxidatively stable in relation to similar ones based on the diphenylethane-derivative chain extender [72]. 

**Figure 12:**
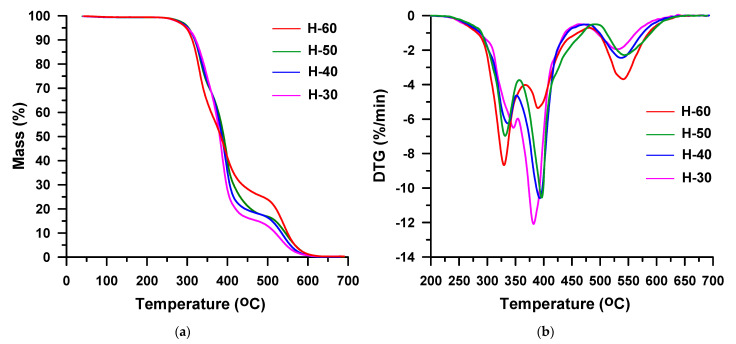
TG (**a**) and DTG (**b**) curves of the HDI-based TPU elastomers obtained in air.

**Figure 13:**
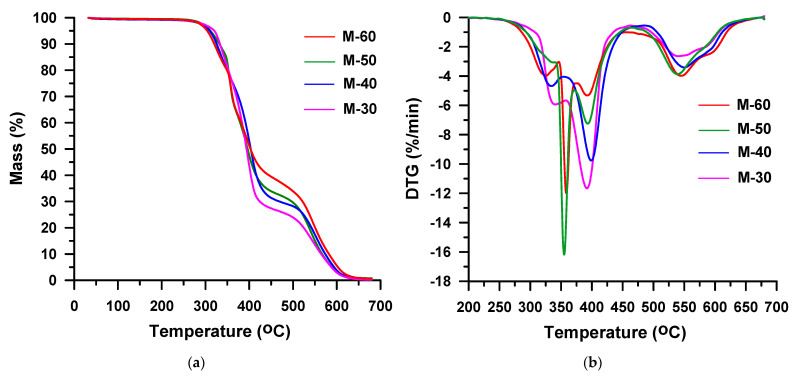
TG (**a**) and DTG (**b**) curves of the MDI-based TPU elastomers obtained in air.

In the air atmosphere all TPU elastomers synthesized from HDI decompose in three stages (Figure 12). On the DTG curves, apart from the peaks observed in helium up to ~500 °C, there is a well-separated one with *T*_max_ = 530–543 °C. This last stage of decomposition is associated with a mass loss of 15 to 25%. A three-stage decomposition is also visible for the MDI-based TPU elastomers with 30 and 40 mas% content of hard segments, while the other two polymers in the series decompose in four steps (Figure 13). For the former polymers, a new peak with *T*_max_ = 540 or 549 °C emerges and the maximum of lower-temperature peak shifts from 364 to 335 °C or from 370 to 340 °C. For the latter, two new peaks with *T*_max_ = 336 or 326 °C and 529 or 545 °C appear on the DTG curves compared to the degradation in helium. With the increase in the content of hard segments, the mass losses connected with the decomposition stage with *T*_max_ = 392–399 °C decrease (from 51 to 24%) and those related to the earlier decomposition stage or stages with *T*_max_ = 335–359 °C (from 23% to 38%) increase. Hence, it can be concluded that, as in a non-oxidizing atmosphere, the hard segments decompose first and only then the soft segments. In addition, the mass losses corresponding to the final stage of decomposition, in which oxidation processes occur, increase from 20% to 38% as the content of hard segments grows.

The decomposition of M-50 sample in air is connected as a rule with elimination of the same type of products as in helium, as shown in Figure 11. Similar situation is observed for the H-50 sample (see Figure 10). Only the isocyanates are not detected, which may be due to their complete carbodiimidization. It can also be seen that more diverse organic carbonyl compounds are released. Absorption bands responsible for the stretching vibrations of C=O bonds are located in the higher range of wavenumbers, i.e., 1790–1701 cm^–1^. The literature shows that the PTMO produces butanal, propanal and ethanal, methanol and carbon dioxide when heated in an oxidizing atmosphere; ethers were not detected [60]. From Figure 10 and Figure 11, it follows that during the decomposition of the studied TPU elastomers ethers are evolved, as they did in inert conditions. The analysis of the FTIR spectra also shows that the decomposition of sulfide bonds (COS emission) occurs for sample H-50 in the first stage, and for sample M-50 in both the first and second stages. The decomposition of polyether soft segments and aromatic content, as in the helium atmosphere, takes place mainly in the stage with a *T*_max_ of about 395 °C.

## 4. Conclusions

The thermal stability of two types of TPU elastomers synthesized from a nonconventional chain extender is relatively good. Nevertheless, polymers derived from aromatic diisocyanate MDI are more resistant to high temperatures than their HDI-based analogs, both in inert and oxidizing atmospheres. In helium, they are stable up to 299–301 °C vs. 280–282 °C, while in synthetic air up to 261–272 °C vs. 252–265 °C, considering a 1% mass loss temperature. Based on this study, it can further be stated that under inert conditions these polymers decompose almost completely up to the temperature of 600 °C, although in the case of those based on MDI the residues are slightly larger (2–6% vs. 1%) and increase with a rising content of hard segments. In turn, in synthetic air, due to the occurrence of oxidative processes, they are fully decomposed. The tested atmosphere also has an effect on the number of decomposition steps for these polymers. In helium, they decompose in two steps (except for the H-30 sample), while in synthetic air in three or four. The hard segments start decomposing first, followed by the polyether soft segments. However, the decomposition of hard segments begins with that of the urethane bonds, not the sulfide ones. The analysis of volatile decomposition products shows that the breakdown of the urethane bonds in TPU elastomers occurs due to their dissociation into alcohols and isocyanates. In addition to an isocyanate (for the TPU elastomers obtained from HDI) and alcohols, carbon dioxide, carbon monoxide, water, carbonyl sulfide, sulfur dioxide, ethers, organic carbonyl compounds, including aldehydes, as well as aromatic and unsaturated aliphatic compounds were found in the volatile decomposition products.

## Data Availability

The data presented in this study are available on request from the author.
